# The Association of Vitamin D Receptor Polymorphisms with Multiple Sclerosis in a Case-Control Study from Kuwait

**DOI:** 10.1371/journal.pone.0142265

**Published:** 2015-11-05

**Authors:** Rabeah Abbas Al-Temaimi, Anwar Al-Enezi, Ahmad Al-Serri, Raed Al-Roughani, Fahd Al-Mulla

**Affiliations:** 1 Human Genetics Unit, Department of Pathology, Faculty of Medicine, Kuwait University, Jabriya, Kuwait; 2 Pathology Master’s Program, Department of Pathology, Faculty of Medicine, Kuwait University, Jabriya, Kuwait; 3 Division of Neurology, Department of Medicine, Amiri Hospital, Kuwait City, Kuwait; 4 Neurology Clinic, Department of Medicine, Dasman Diabetes Institute, Kuwait City, Kuwait; 5 Molecular Pathology Unit, Department of Pathology, Faculty of Medicine, Kuwait University, Jabriya, Kuwait; University of Oxford, UNITED KINGDOM

## Abstract

Vitamin D deficiency is associated with several diseases including multiple sclerosis (MS). Several factors influence vitamin D levels and its optimal multi-function maintenance. Our objective was to assess quantifiable variables influencing vitamin D level and metabolism in MS patients from Kuwait. In a case-control study involving 50 MS patients, and 50 healthy control individuals for which plasma vitamin D levels, supplement use, vitamin D receptor (*VDR*) variants, and skin pigmentation indices were ascertained; we found overall vitamin D levels to be deficient in both groups, and supplement use to be common practice. VDR variants *Taq*I and *Bsm*I associated with MS risk, and *Apa*I associated with low disease progression. VDR variant *Fok*I associated with higher vitamin D levels in both groups. We conclude that several quantifiable variables related to vitamin D associate with MS suggesting a possible clinical immuno-modulatory application of vitamin D for MS patients in Kuwait.

## Introduction

Multiple Sclerosis (MS) is a chronic, autoimmune neurodegenerative disease of the central nervous system. MS is considered a complex disorder that has been suggested to result from interplay of environmental and genetic factors predisposing to disease risk. Vitamin D deficiency has been associated with increased risk and progression of several diseases [1], including (MS) [2–5]. The prevalence of MS is highest where environmental supplies of vitamin D are lowest [6]. Kuwait has become a high-risk area for MS; its prevalence has increased from 4.4 in 1990 to 85 cases per 100,000 individuals in 2014 [7, 8]. Kuwait is located between latitudes of 28.45 and 30.05 degrees north of the Equator, where ample solar UV radiation could allow for optimal vitamin D synthesis in the skin. Despite our location levels of vitamin D levels have been reported to be deficient in Kuwait, possibly due to several factors that play a role in vitamin D synthesis and ideal use in the body [9]. Such factors include skin pigmentation and age, levels of 7-dehydrocholesterol (pro-vitamin D3), individual sun exposure duration and time of day, amount of unprotected skin exposed to UV radiation, body’s inherent genetic factors influencing adequate synthesis and function, and underlying physiological conditions affecting vitamin D metabolism and functions [10]. Dietary sources such as fish consumption and supplements; also contribute to vitamin D levels and can be considered accessory to skin vitamin D synthesis.

Vitamin D has several functions that are critical for a healthy body. Vitamin D is essential for bone and calcium homeostasis, adequate neuromuscular functions, brain neurotransmission and detoxification pathways, and immune system regulation and maintenance [11–14]. Vitamin D mediates its function through the ubiquitously expressed vitamin D receptor (*VDR*, GeneID: 7421). Reported evidence from many studies on *VDR* single nucleotide polymorphisms (SNPs) associating with disease singles out four SNPs of possible disease association; three at the 3' end of the *VDR* gene (*Taq*I, *Bsm*I, *Apa*I) and one at the 5’end (*Fok*I) [15]. The association of these SNPs with MS is controversial, and is suggested to be influenced by study characteristics and ethnicity [16, 17]. Here we set out to determine vitamin D related quantitative variables association with MS risk in Kuwait; inclusive of vitamin D levels, vitamin D supplementation, *VDR* polymorphisms, and sun exposure. We found vitamin D levels to be deficient in our sampled population, and *VDR* genetic variants to associate with several aspects of MS.

## Materials and Methods

### Case-control cohorts sample collection

This study involved 50 Kuwaiti MS patients and 50 healthy control individuals who were age and sex matched for a case-control study design. Patients were recruited at the MS clinic at Dasman Diabetes Institute (DDI). Information of procedures to be performed was fully explained to patients prior to procurement of their informed consent in agreement with the Joint Committee for The Protection of Human Subjects at Kuwait’s health sciences center (HSC), and in agreement with the ethical review committee (ERC) of DDI. Patients were recruited based on availability of a detailed clinical history (demographics, age of onset, disease duration, expanded disability status scale (EDSS) score, and treatments received), being born in Kuwait and have resided in Kuwait from birth to at least early adult life, and the willingness to provide a 3–4 ml blood sample. Cohorts’ demographics and clinical characteristics are shown in Table 1. Participants in this study were asked about their vitamin D supplement intake and type of supplements used to better assess the contribution of supplement on vitamin D levels in plasma. Progression index (PI) was computed for each patient to determine the rate of disease progression by dividing EDSS score over disease duration since diagnosis. Patients were grouped into PI low if PI ≤ 0.5, and intermediate to high if PI ≥ 0.51 [18]. HLA-DRB1*51 status for both cohorts were previously determined by rs3135005 genotyping [19].

**Table 1:** Cohorts’ demographics, clinical characteristics and vitamin D levels and supplement use.

Criteria		MS patients (n = 50)	Healthy controls (n = 50)
Sex [n (%)]	Female	33 (66)	31 (62)
	Male	17 (34)	19 (38)
Age	Mean in years, ±SD	33.44 ±9.63	28.68 ±7.98
	range	18–54	17–60
MS type	RRMS/SPMS	48/2	-
Disease duration	Mean in years, ±SD	8.2 ±5.11	-
	range	1–23	-
EDSS	Median	3	-
	IQR	2.5–4.5	-
PI	Median	0.5	-
	IQR	0.3175–0.83	-
	Low, n	32	-
	High, n	18	-
HLA-DRB1*51 status	Positive	14	16
	Negative	36	34
Vitamin D level	Median (ng/mL)	12.68	11.52
	range	3.9–46.4	0.6–28.6
Vitamin D status	Deficiency	38	41
	Insufficiency	9	9
	Optimal	3	0
Vitamin D supplement	Yes	8	26
	No	42	24

### Vitamin D assay

Blood samples were centrifuged at 2,500xg for 5 minutes in a swinging bucket centrifuge at room temperature to separate blood phases. Isolated plasma fractions were subjected to an enzyme linked immuno-sorbent assay (ELISA) to assess plasma circulating 25-hydroxyvitamin D (25-OH-VitD) levels (the sum of 25-OH-vitamin D2 and 25-OH-vitamin D3) using Eagle Biosciences’ 25-OH vitamin D ELISA kit (Eagle Biosciences, NH, USA). In summary, the assay included 6 standards and 2 controls for deficient and optimal vitamin D levels, respectively. Standards and controls were prepared for a final volume of 200μl according to manufacturer’s protocol, whereas 20μl of EDTA-plasma samples were diluted in 1%biotin/sample buffer for a 200μl volume of sample per well. The 96-well plate was pre-coated with monoclonal antibody specific for both vitamin D2 and vitamin D3 at 100% specificity. The samples were allowed to incubate for two hours at room temperature, followed by 3 rigorous washes with 300μl of wash buffer. The wells were dried and 100μl of enzyme conjugate were added to each well and the plate was incubated for 30 minutes at room temperature. Following incubation three washes with 300μl of wash buffer were performed and the plate was dried prior to the addition of 100μl of substrate solution. The substrate was incubated at room temperature for 15 minutes in the dark. Following incubation, 100μl of stop solution were added and color development was measured photo- metrically at 450 nm wavelength, and a reference wavelength between 620 nm and 650 nm within 30 minutes of adding the stop solution. A standard curve with a 0–120 ng/ml range was plotted and unknown samples’ vitamin D levels were ascertained. Vitamin D levels were assigned into groups of deficient (<20 ng/mL), vitamin D insufficient (21–29 ng/mL), and vitamin D optimal (30–85 ng/mL), in accord with The Endocrine Society’s Clinical Guidelines [20].

### *VDR* SNPs genotyping assays

Approximately 3–4 ml of blood was collected from each individual and an aliquot of 200μl of blood was retained for DNA extraction prior to blood fractionation. Genomic DNA was extracted using QIAamp DNA blood mini kit (Qiagen, CA, USA). Manufacturer protocol was followed with minor modifications. In brief, whole blood samples were added to 25μl of proteinase K and 200μl of lysis buffer. Incubation at 56°C was increased to 30 minutes to maximize lysis. DNA was precipitated in 200μl absolute ethanol and bound to a filtered spin column. The column was washed twice in two different concentrations of wash buffer, followed by a drying spin at maximum speed for 1 minute. DNA was eluted with nuclease free water and DNA yield and quality were determined using a NanoDrop spectrophotometer. Four common *VDR* SNPs were selected based on their variable associations with a wide array of pathological conditions and physiological states. Those SNPs are commonly referred to by their specific restriction enzymes when assayed using Restriction Fragment Length Polymorphism assays, they are *Taq*I (rs731236), *Bsm*I (rs1544410), *Fok*I (rs2228570), and *Apa*I (rs7975232). Taqman genotyping assays (Life Technologies, CA, USA) for all SNPs were used for genotyping according to standard protocols and analyzed using ABI 7500 Fast Real-time PCR system (Life technologies, CA, USA).

### Skin pigmentation assessment

Skin melanin and erythema (Hemoglobin) indices are two factors that influence vitamin D synthesis in the skin [21]. Using a hand-held spectrohphotometer (DermaSpectroMeter (DSM II) colorMeter, MIC Global, London, UK) the two pigments were measured from two skin areas. Two measurements were recorded for constitutive skin color which describes the genetically determined level of melanin in the skin that is not influenced by environmental factors. And two measurements were recorded for facultative pigmentation that shows changes in skin pigmentation as a result of environmental factors. Constitutive skin pigmentation was measured from upper inner arms, where there is limited sun exposure. Whereas, facultative pigmentation was measured from outside of the lower arm, or forehead depending on whether tanning or sun protection products were applied by the participant. Any skin area with coatings, moles, scars, or excess hair were avoided to ensure uniform skin measurements. Individuals who use tanning booths were excluded from this study. All measurements were conducted between the months of February-May during spring season where most individuals in Kuwait would engage in outdoor activities.

### Statistical analyses

Statistical analyses were performed using GraphPad prism 6.0 software (GraphPad Software, Inc., CA, USA), and SPSS software (version 22; SPSS Inc, Chicago, IL, USA). None of the ELISA results were normally distributed so a Mann-Whitney rank sum test was used to compare groups with a two-tailed significance of p < 0.05. Odds ratio (OR) was used at 95% confidence interval (CI) for differences between groups comparisons, and p < 0.05 was considered significant. In addition, Bonferroni corrected (p < 0.0083) tests of association including; Fisher exact test, Chi-square test, and Student t-test were used when appropriate for the effect of each *VDR* SNP on MS risk, clinical course, and vitamin D levels. Analysis of variance (ANOVA) was used for co-variate analyses of *VDR* haplotype.

## Results

### Vitamin D levels in study cohorts

Vitamin D levels were overall deficient and below the optimal levels recommended for healthy physiological vitamin D functions (Table 1). There were 38 (76%) MS patients with vitamin D deficiency, 9 (18%) had vitamin D insufficiency, and 3 (6%) patients had optimal vitamin D levels. In healthy controls there were 41 (82%) individuals with deficient vitamin D levels, and 9 (18%) with insufficient vitamin D levels. There were no healthy controls with optimal vitamin D levels. Combining both cohorts resulted in an overall vitamin D deficiency in 79% and 18% insufficiency suggesting vitamin D levels might be deficient in a major subset of the Kuwaiti population. There were no significant differences in vitamin D levels between MS and healthy control individuals. Considering the effect of vitamin D supplement, we found that 52% of healthy controls were taking vitamin D supplement in the form of tablets (61.54%), or intravenous injections (19.23%), or both forms of supplementation (19.23%). Whereas only 30% of MS patients were taking some type of supplements, only 53% of them listed vitamin D as a supplement and all in the form of tablet. There were no significant differences between vitamin D levels among healthy controls on supplement and those without supplement (unpaired t-test; p = 0.09). Similarly, vitamin D supplementation did not affect vitamin D levels among MS patients. Comparing MS patients to healthy controls vitamin D levels with or without supplementation were not significant. We then examined whether vitamin D levels associated with EDSS score or disease progression index (PI) in MS patients. We found no significant association between disease clinical disability assessments and vitamin D levels.

### Effects of *VDR* SNPs on vitamin D levels and MS risk

*VDR* SNPs genotype and allelic distribution are shown in Table 2. Each SNP was analyzed separately for its association with MS risk and study variables. *Taq*I (rs731236) genotype distribution was significantly different in MS patients than healthy controls (2x3 Chi square; p = 0.0008), with allele G associating with MS risk (2x2 Chi square; p = 0.0007, OR 3.087, 95%CI: 1.63–5.84). However, *Taq*I genotypes had no influence on vitamin D levels, vitamin D status groups, EDSS or PI in MS patients. Similarly, *Bsm*I (rs1544410) genotype distribution differed significantly in MS patients than healthy controls (2x3 Chi square; p = 0.003), with the C allele associating with MS risk (2x2 Chi square, p = 0.028, OR 1.91, 95%CI: 1.067–3.4). No significant associations were found for *Apa*I genotypes distribution when comparing vitamin D levels, vitamin D status, or EDSS of MS patients. *Apa*I (rs7975232) genotype distribution did not differ between MS and healthy control individuals, however *Apa*I genotype AA associated significantly with low PI (2x3 Chi square, p = 0.003). Using an additive genotype model (AC+CC) further confirmed the association of genotype AA with low PI (2x2 Chi square; p = 0.0028, OR 0.12, 95%CI: 0.028–0.502). *Fok*I (rs2228570) genotypes did not associate with MS risk or clinical characteristics, and genotype GG did not reach adjusted significance for its effect on plasma vitamin D levels in MS patients not on supplement (Mann-Whitney test; p = 0.013). None of the SNPs assayed had a profound influence on response to supplement when comparing genotypes to vitamin D levels of individuals on supplement. In addition, *VDR* SNPs haplotype analysis did not reveal any significant association.

**Table 2:** *VDR* SNPs genotype distributions in our study cohorts.

*VDR* SNPs genotypes [n (%)]		MS patients	Healthy controls
*Taq*I	GG	31 (62)	15 (30)
	GA	19 (38)	28 (56)
	AA	0 (0)	7 (15)
*Bsm*I	CC	20 (40)	15 (30)
	CT	30 (60)	25 (50)
	TT	0 (0)	10 (20)
*Apa*I	AA	20 (40)	23 (46)
	AC	25 (50)	17 (34)
	CC	5 (10)	10 (20)
*Fok*I	GG	33 (66)	33 (66)
	AG	14 (28)	16 (32)
	AA	3 (6)	1 (2)

### Skin pigmentation indices of study cohorts

Comparing constitutive melanin indices of MS patients against healthy controls did not show any association (Table 3). Vitamin D levels did not show any significant relation to constitutive melanin indices; neither did facultative melanin, constitutive erythema, or facultative erythema indices. However, facultative melanin indices were significantly higher in MS males than MS females (Mann-Whitney test; p = 0.002). A finding that was also found in healthy control males compared to healthy control females albeit with reduced significance (Mann-Whitney test; p = 0.035). No differences were seen when comparing healthy males to MS males’ facultative melanin indices. Moreover, facultative erythema was higher in MS males compared to MS females (Mann-Whitney test; p = 0.0006), and in healthy male controls compared to healthy female controls (Mann-Whitney test; p = 0.0008). No differences were found when comparing healthy males to MS males’ facultative erythema indices. Calculated change in melanin index due to sun exposure (Difference in melanin) was higher among MS patients than healthy controls (Unpaired t-test; p = 0.0084), MS females had higher values compared to healthy females (Unpaired t-test; p = 0.028), and MS males had higher values compared to healthy males (Unpaired t-test; p = 0.034). The major contributor to difference in melanin in our MS cohort was the MS males as their difference in melanin was significantly higher than MS females (Unpaired t-test, p = 0.0007). This data indicates that MS males have the most sun exposure evaluated by skin melanin content changes and erythema; than other sex segregated groups in our cohorts. None of the studied *VDR* SNPs genotypes segregated with facultative melanin, facultative erythema, constitutive erythema, or differences in melanin or erythema.

**Table 3:** Skin pigmentation measurements from our study cohorts included constitutive melanin and erythema, and facultative melanin and erythema indices. Difference in melanin indices were used to estimate sun exposure, and difference in erythema to determine skin sensitivity to sun burns.

Skin measurement indices		MS patients	Healthy controls
Constitutive melanin	Mean ± SD	35.9 ± 4.1	37.03 ± 4.9
	range	27.2–50.7	26.5–51.6
Facultative melanin	Mean ± SD	41.4 ± 6.9	40.0 ± 6.6
	Range	30.4–63.0	27.7–57.4
Constitutive erythema	Mean ± SD	10.3 ± 2.5	12.0 ± 3.1
	Range	5.1–15.3	6.9–19.1
Facultative erythema	Mean ± SD	13.8 ± 2.8	15.3 ± 3.2
	Range	7.1–20.7	9.1–22.4
Melanin difference	Mean ± SD	5.5 ± 4.8*	2.9 ± 4.1
Erythema difference	Mean ± SD	3.5 ± 2.3	3.2 ± 2.0

*Denotes significant difference of p-value <0.05.

## Discussion

Assessing quantifiable variable influencing vitamin D levels in MS patients, we found both healthy controls and MS patients to be deficient of optimal vitamin D levels. Although both patients and healthy controls are aware of the importance of vitamin D as indicated by their use of supplement; supplement intake did not affect their vitamin D levels. There are several factors that might be contributing to this outcome which include sub-optimal vitamin D supplement dosage, infrequent supplementation, deficiencies in complementary vitamin D sources such as diet and sun exposure, or/and genetic predisposition to inadequate vitamin D synthesis and metabolism. Vitamin D supplement dosage and frequency were unquantifiable as both cohorts had difficulty recollecting this information. Sun exposure was assessed and showed that females had a lower sun exposure than males, with male MS patients showing the highest differences in skin melanin content in response to sun exposure. However, MS males’ sun exposure did not influence their vitamin D levels suggesting other factors, possibly genetic; are involved. This suggests that female MS patients are having inadequate sun exposure. Inadequate female sun exposure is possibly affected by choice of clothing and life style factors including religious belief and traditional choices of clothing. In addition to some psychological factors expressed by some patients that limit their willingness to partake in outdoor physical activities.

The *VDR* gene is the most widely studied genetic factor implicated in vitamin D deficiency. We selected four *VDR* SNPs for their association with MS and found that each associated with a different outcome in assayed variables. *Taq*I and *Bsm*I associated with MS risk in our cohort. *Taq*I genotype GG has been controversially linked to lower *VDR* expression [22]. It is plausible that lower *VDR* expression results in low vitamin D utilization affecting the efficiency of its downstream pathways and functions in MS patients. Although our finding is in agreement with other studies from Iran [23, 24], Tunisia [25], Australia [26], and Japan [27]; studies from European Caucasian populations have often disputed these associations in their MS populations [28–31]. There is a clear indication that ethnicity is the basis for the discrepancy in *VDR*’s *Taq*I and *Bsm*I SNPs association with MS; possibly in addition to other co-segregating genetic factors. For example Agliardi *et al*. reported *Taq*I AA genotype affect is only evident when co-segregating with HLA-DRB1*15 positive haplotype in MS patients [32]. However, in our MS cohort HLA-DRB1*15 haplotype is not associated with MS risk, and does not influence *Taq*I association with MS risk (data not shown). *Apa*I did not associate with MS risk in our cohort, although it was reported in the above mentioned populations to also associate with MS risk. In our MS cohort *Apa*I genotype AA associated with slow disease progression, a finding that needs to be replicated in a larger MS cohort. It is unclear how *Apa*I AA genotype results in slower progression, since *Apa*I AA genotype was also shown to associate with less stable VDR expression and lower bone mineral density in the lumbar spine; both of which directly affect vitamin D levels and calcium homeostasis [33, 34].

The association of *Fok*I with slightly higher vitamin D levels is contradictory. GG genotype associating with higher skin constitutive melanin content has been reported previously in a South African study, albeit without a significant correlation to vitamin D or VDR levels [35]. *Fok*I GG genotype results in a smaller VDR isoform; that influences VDR transactivation capacity in a gene specific manner [36, 37]. The association of GG genotype with higher vitamin D levels is novel in our study, as a previous meta-analysis study found individuals of lower skin pigmentation to have higher vitamin D levels [38]. However, the effect of other factors influencing vitamin D levels was not included in that study. Other factors such as effects of culture on clothing style, time spent outdoors, sun protection habits, vitamin D intake from diet, use of supplements, and parathyroid hormone levels along with other genetic factors were not considered. In addition, Smolders *et al*., reported an association of *Fok*I genotype GG in MS patients with higher bioactive vitamin D (calcitriol) levels but not 25(OH)D [39]. It is possible that our ELISA assay has a limitation that resulted in cross-reactivity with bioactive calcitriol contributing to this association. In summary, it is unclear how the functional attributes of GG genotype influence vitamin D levels but its association with increased skin pigmentation might offer some insight. It is plausible that increased constitutive melanin allows for longer sun exposure tolerance and higher skin vitamin D synthesis in GG genotype group at a fixed latitude.

In conclusion, we have assayed quantifiable modifiers of vitamin D levels in an MS case-control study and found associations of *VDR* SNPs with MS risk, disability progression, and vitamin D levels. Although our findings need to be replicated in a larger cohort to guide applied clinical significance; it is possible that optimal vitamin D levels throughout the year may be beneficial for MS patients, both to obtain immune-mediated suppression of disease activity, and to decrease disease-related complications.

## References

[1] Autier P, Boniol M, Pizot C, Mullie P. Vitamin D status and ill health: a systematic review. Lancet Diabetes Endocrinol. 2014 1;2(1):76–89. Epub 2014/03/14. eng. 10.1016/S2213-8587(13)70165-7 24622671

[2] Munger KL, Kochert K, Simon KC, Kappos L, Polman CH, Freedman MS, et al Molecular mechanism underlying the impact of vitamin D on disease activity of MS. Ann Clin Transl Neurol. 2014 8;1(8):605–17. Pubmed Central PMCID: 4180413. Epub 2014/10/07. eng. 10.1002/acn3.91 25285313 PMC4180413

[3] Ascherio A, Munger KL, White R, Kochert K, Simon KC, Polman CH, et al Vitamin D as an early predictor of multiple sclerosis activity and progression. JAMA Neurol. 2014 3;71(3):306–14. Pubmed Central PMCID: 4000029. Epub 2014/01/22. eng. 10.1001/jamaneurol.2013.5993 24445558 PMC4000029

[4] Martinelli V, Dalla Costa G, Colombo B, Dalla Libera D, Rubinacci A, Filippi M, et al Vitamin D levels and risk of multiple sclerosis in patients with clinically isolated syndromes. Mult Scler. 2014 2;20(2):147–55. Epub 2013/07/10. eng. 10.1177/1352458513494959 23836877

[5] Antico A, Tampoia M, Tozzoli R, Bizzaro N. Can supplementation with vitamin D reduce the risk or modify the course of autoimmune diseases? A systematic review of the literature. Autoimmun Rev. 2012 12;12(2):127–36. Epub 2012/07/11. eng. 10.1016/j.autrev.2012.07.007 22776787

[6] VanAmerongen BM, Dijkstra CD, Lips P, Polman CH. Multiple sclerosis and vitamin D: an update. Eur J Clin Nutr. 2004 8;58(8):1095–109. . Epub 2004/04/01. eng. 15054436 10.1038/sj.ejcn.1601952

[7] al-Din AS, Khogali M, Poser CM, al-Nassar KE, Shakir R, Hussain J, et al Epidemiology of multiple sclerosis in Arabs in Kuwait: a comparative study between Kuwaitis and Palestinians. Journal of the neurological sciences. 1990 12;100(1–2):137–41. . 2089130 10.1016/0022-510x(90)90024-h

[8] Alroughani R, Ahmed SF, Behbahani R, Khan R, Thussu A, Alexander KJ, et al Increasing prevalence and incidence rates of multiple sclerosis in Kuwait. Multiple sclerosis. 2014 4;20(5):543–7. 10.1177/1352458513504328 24025709

[9] Fields J, Trivedi NJ, Horton E, Mechanick JI. Vitamin D in the Persian Gulf: integrative physiology and socioeconomic factors. Curr Osteoporos Rep. 2011 12;9(4):243–50. Epub 2011/09/09. eng. 10.1007/s11914-011-0071-2 21901427

[10] Webb AR. Who, what, where and when-influences on cutaneous vitamin D synthesis. Prog Biophys Mol Biol. 2006 9;92(1):17–25. . Epub 2006/06/13. eng. 16766240 10.1016/j.pbiomolbio.2006.02.004

[11] Stocklin E, Eggersdorfer M. Vitamin D, an essential nutrient with versatile functions in nearly all organs. Int J Vitam Nutr Res. 2013;83(2):92–100. Epub 2014/02/05. eng. 10.1024/0300-9831/a000151 24491882

[12] Harms LR, Burne TH, Eyles DW, McGrath JJ. Vitamin D and the brain. Best Pract Res Clin Endocrinol Metab. 2011 8;25(4):657–69. Epub 2011/08/30. eng. 10.1016/j.beem.2011.05.009 21872806

[13] Lang PO, Aspinall R. Can we translate vitamin D immunomodulating effect on innate and adaptive immunity to vaccine response? Nutrients. 2015 3;7(3):2044–60. Pubmed Central PMCID: 4377899. Epub 2015/03/25. eng. 10.3390/nu7032044 25803545 PMC4377899

[14] Fernandes de Abreu DA, Eyles D, Feron F. Vitamin D, a neuro-immunomodulator: implications for neurodegenerative and autoimmune diseases. Psychoneuroendocrinology. 2009 12;34 Suppl 1:S265–77. Epub 2009/06/24. eng. 10.1016/j.psyneuen.2009.05.023 19545951

[15] Uitterlinden AG, Fang Y, van Meurs JB, van Leeuwen H, Pols HA. Vitamin D receptor gene polymorphisms in relation to Vitamin D related disease states. J Steroid Biochem Mol Biol. 2004 5;89-90(1–5):187–93. . Epub 2004/07/01. eng. 15225770 10.1016/j.jsbmb.2004.03.083

[16] Huang J, Xie ZF. Polymorphisms in the vitamin D receptor gene and multiple sclerosis risk: a meta-analysis of case-control studies. J Neurol Sci. 2012 2 15;313(1–2):79–85. Epub 2011/10/28. eng. 10.1016/j.jns.2011.09.024 22029942

[17] Tizaoui K, Kaabachi W, Hamzaoui A, Hamzaoui K. Association between vitamin D receptor polymorphisms and multiple sclerosis: systematic review and meta-analysis of case-control studies. Cell Mol Immunol. 2015 3;12(2):243–52. Epub 2014/07/08. eng. 10.1038/cmi.2014.47 24998351 PMC4654294

[18] Laaser U, Senault R, Viefhues H, Deutsche Gesellschaft für Sozialmedizin., Deutsches Institut zur Bekämpfung des Hohen Blutdruckes. Primary health care in the making Berlin; New York: Springer-Verlag; 1985 xxiv, 545 p. p.

[19] Al-Temaimi R, Alroughani R, Jacob S, Al-Mulla F. Gender influence in EBV antibody response in multiple sclerosis patients from Kuwait. Journal of neuroimmunology. 2015 8 15;285:57–61. 10.1016/j.jneuroim.2015.05.021 26198919

[20] Holick MF, Binkley NC, Bischoff-Ferrari HA, Gordon CM, Hanley DA, Heaney RP, et al Evaluation, treatment, and prevention of vitamin D deficiency: an Endocrine Society clinical practice guideline. The Journal of clinical endocrinology and metabolism. 2011 7;96(7):1911–30. 10.1210/jc.2011-0385 21646368

[21] Nessvi S, Johansson L, Jopson J, Stewart A, Reeder A, McKenzie R, et al Association of 25-hydroxyvitamin D3)levels in adult New Zealanders with ethnicity, skin color and self-reported skin sensitivity to sun exposure. Photochem Photobiol. 2011 Sep-Oct;87(5):1173–8. Epub 2011/06/18. eng. 10.1111/j.1751-1097.2011.00956.x 21679191

[22] Durrin LK, Haile RW, Ingles SA, Coetzee GA. Vitamin D receptor 3'-untranslated region polymorphisms: lack of effect on mRNA stability. Biochim Biophys Acta. 1999 3 30;1453(3):311–20. . Epub 1999/04/02. eng. 10101249 10.1016/s0925-4439(99)00007-1

[23] Narooie-Nejad M, Moossavi M, Torkamanzehi A, Moghtaderi A, Salimi S. Vitamin D Receptor Gene Polymorphism and the Risk of Multiple Sclerosis in South Eastern of Iran. J Mol Neurosci. 2015 2 18 . Epub 2015/04/10. Eng. 25854779 10.1007/s12031-015-0513-x

[24] Narooie-Nejad M, Moossavi M, Torkamanzehi A, Moghtaderi A. Positive association of vitamin D receptor gene variations with multiple sclerosis in South East Iranian population. Biomed Res Int. 2015;2015:427519 Pubmed Central PMCID: 4320885. Epub 2015/02/17. eng. 10.1155/2015/427519 25685788 PMC4320885

[25] Ben-Selma W, Ben-Fredj N, Chebel S, Frih-Ayed M, Aouni M, Boukadida J. Age- and gender-specific effects on VDR gene polymorphisms and risk of the development of multiple sclerosis in Tunisians: a preliminary study. Int J Immunogenet. 2015 4 20. 10.1111/iji.12197 25892553

[26] Tajouri L, Ovcaric M, Curtain R, Johnson MP, Griffiths LR, Csurhes P, et al Variation in the vitamin D receptor gene is associated with multiple sclerosis in an Australian population. J Neurogenet. 2005 Jan-Mar;19(1):25–38. . Epub 2005/08/04. eng. 16076630 10.1080/01677060590949692

[27] Fukazawa T, Yabe I, Kikuchi S, Sasaki H, Hamada T, Miyasaka K, et al Association of vitamin D receptor gene polymorphism with multiple sclerosis in Japanese. J Neurol Sci. 1999 6 15;166(1):47–52. . Epub 1999/08/28. eng. 10465499 10.1016/s0022-510x(99)00112-4

[28] Dickinson JL, Perera DI, van der Mei AF, Ponsonby AL, Polanowski AM, Thomson RJ, et al Past environmental sun exposure and risk of multiple sclerosis: a role for the Cdx-2 Vitamin D receptor variant in this interaction. Mult Scler. 2009 5;15(5):563–70. Epub 2009/04/23. eng. 10.1177/1352458509102459 19383647

[29] Smolders J, Damoiseaux J, Menheere P, Tervaert JW, Hupperts R. Association study on two vitamin D receptor gene polymorphisms and vitamin D metabolites in multiple sclerosis. Ann N Y Acad Sci. 2009 9;1173:515–20. Epub 2009/09/18. eng. 10.1111/j.1749-6632.2009.04656.x 19758194

[30] Sioka C, Papakonstantinou S, Markoula S, Gkartziou F, Georgiou A, Georgiou I, et al Vitamin D receptor gene polymorphisms in multiple sclerosis patients in northwest Greece. J Negat Results Biomed. 2011;10:3 Pubmed Central PMCID: 3097159. Epub 2011/05/07. eng. 10.1186/1477-5751-10-3 21545713 PMC3097159

[31] Garcia-Martin E, Agundez JA, Martinez C, Benito-Leon J, Millan-Pascual J, Calleja P, et al Vitamin D3 receptor (VDR) gene rs2228570 (Fok1) and rs731236 (Taq1) variants are not associated with the risk for multiple sclerosis: results of a new study and a meta-analysis. PLoS One. 2013;8(6):e65487 Pubmed Central PMCID: 3688728. Epub 2013/07/11. eng. 10.1371/journal.pone.0065487 23840333 PMC3688728

[32] Agliardi C, Guerini FR, Saresella M, Caputo D, Leone MA, Zanzottera M, et al Vitamin D receptor (VDR) gene SNPs influence VDR expression and modulate protection from multiple sclerosis in HLA-DRB1*15-positive individuals. Brain Behav Immun. 2011 10;25(7):1460–7. Epub 2011/06/15. eng. 10.1016/j.bbi.2011.05.015 21664963

[33] Uitterlinden AG, Fang Y, Van Meurs JB, Pols HA, Van Leeuwen JP. Genetics and biology of vitamin D receptor polymorphisms. Gene. 2004 9 1;338(2):143–56. . Epub 2004/08/19. eng. 15315818 10.1016/j.gene.2004.05.014

[34] Christensen MH, Apalset EM, Nordbo Y, Varhaug JE, Mellgren G, Lien EA. 1,25-dihydroxyvitamin D and the vitamin D receptor gene polymorphism Apa1 influence bone mineral density in primary hyperparathyroidism. PLoS One. 2013;8(2):e56019 Pubmed Central PMCID: 3572149. Epub 2013/02/19. eng. 10.1371/journal.pone.0056019 23418495 PMC3572149

[35] V ON, Asani FF, Jeffery TJ, Saccone DS, Bornman L. Vitamin D Receptor Gene Expression and Function in a South African Population: Ethnicity, Vitamin D and I. PLoS One. 2013;8(6):e67663 . Pubmed Central PMCID: 3689684. Epub 2013/06/28. Eng. 23805323 10.1371/journal.pone.0067663 PMC3689684

[36] Whitfield GK, Remus LS, Jurutka PW, Zitzer H, Oza AK, Dang HT, et al Functionally relevant polymorphisms in the human nuclear vitamin D receptor gene. Mol Cell Endocrinol. 2001 5 25;177(1–2):145–59. . Epub 2001/05/30. eng. 11377830 10.1016/s0303-7207(01)00406-3

[37] Alimirah F, Peng X, Murillo G, Mehta RG. Functional significance of vitamin D receptor FokI polymorphism in human breast cancer cells. PLoS One. 2011;6(1):e16024 Pubmed Central PMCID: 3025916. Epub 2011/02/02. eng. 10.1371/journal.pone.0016024 21283672 PMC3025916

[38] Hagenau T, Vest R, Gissel TN, Poulsen CS, Erlandsen M, Mosekilde L, et al Global vitamin D levels in relation to age, gender, skin pigmentation and latitude: an ecologic meta-regression analysis. Osteoporos Int. 2009 1;20(1):133–40. Epub 2008/05/07. eng. 10.1007/s00198-008-0626-y 18458986

[39] Smolders J, Damoiseaux J, Menheere P, Tervaert JW, Hupperts R. Fok-I vitamin D receptor gene polymorphism (rs10735810) and vitamin D metabolism in multiple sclerosis. J Neuroimmunol. 2009 2 15;207(1–2):117–21. Epub 2009/01/31. eng. 10.1016/j.jneuroim.2008.12.011 19178954

