# Correction: The Association of Vitamin D Receptor Polymorphisms with Multiple Sclerosis in a Case-Control Study from Kuwait

**DOI:** 10.1371/journal.pone.0144565

**Published:** 2015-12-02

**Authors:** The PLOS ONE Staff

The fourth author’s name is spelled incorrectly. The correct name is: Raed Alroughani.

The correct citation is: Al-Temaimi RA, Al-Enezi A, Al-Serri A, Alroughani R, Al-Mulla F (2015) The Association of Vitamin D Receptor Polymorphisms with Multiple Sclerosis in a Case-Control Study from Kuwait. PLoS ONE 10(11): e0142265. doi:10.1371/journal.pone.0142265

The publisher apologizes for the error.
